# Uveitis in the Aging Eye: Incidence, Patterns, and Differential Diagnosis

**DOI:** 10.1155/2015/509456

**Published:** 2015-05-18

**Authors:** Marwan R. Abdulaal, Bachir H. Abiad, Rola N. Hamam

**Affiliations:** Department of Ophthalmology, American University of Beirut Medical Center, P.O. Box 11-0236/D41, Riad El Solh, Beirut 11072020, Lebanon

## Abstract

Uveitis is a vision threatening inflammation of the eye that carries considerable morbidity. It is responsible for 10% of legal blindness in the United States and up to 25% in the developing world. Uveitis in patients more than 60 years of age is less common. The aging body has a changing response of the immune system, which might reflect a different pattern of uveitis in the elderly population. In this paper we review the incidence and patterns of uveitis in the elderly as reported in the literature and discuss changes with time. We also delineate a thorough differential diagnosis of de novo uveitis in the elderly.

## 1. Introduction

Uveitis is inflammation of the middle-lining layer of the eye, comprising the iris, ciliary body, and choroid. It may involve other adjacent tissues, such as the retina, optic nerve, and vitreous humor [1]. This disease is a sight threatening condition worldwide. It accounts for up to 10% of legal blindness in United States and about 25% in the developing countries [2, 3].

This disease is considered to affect young patients with median age at presentation in the third and fourth decade. This impression is based on epidemiology studies published in the 1960s, which demonstrated that uveitis occurs mainly in young adults at 20 to 50 years of age [1]. On the other hand, more recent reports from the United States showed significant increase in number of uveitis cases among elderly patients [4, 5].

According to World Population Aging report (2009), elderly or aged people are persons of 60 years of age or more [6]. Globally the population of older persons is growing considerably faster than the population as a whole, and it is expected to continue growing more rapidly than other age groups at least till 2050 [6]. Taking that into consideration, our knowledge of the prevalence of uveitis and its common types among elderly patients is essential to formulate and evaluate goals and programs and to enhance our understanding of this disease and prevent its long term complications.

The aim of our paper is to review uveitis epidemiology studies among elderly patients. Furthermore, we aim to discuss the pattern of presentation and diagnosis among elderly patients with uveitis and analyze any change over the past 5 decades. We also aim to delineate a thorough differential diagnosis of de novo uveitis in the elderly.

## 2. Methods

We performed an extensive literature search using the MEDLINE database, from 1964 to 2014. The search subject included uveitis: epidemiology, uveitis among elderly, etiology, classification, and diagnosis. The search was limited to the literature pertaining to humans, with no language limitation. Cross-referencing was also performed from the literature examined. An arbitrary method was used to select the studies to be included in our review, the emphasis being placed on obtaining studies that were representative of each region in different time era.

Data pertaining to age, gender, location of uveitis, chronicity, and diagnosis were extracted and analyzed in accordance with the International Uveitis Study Group (IUSG) recommendations [7]. Data from studies including adequate patient numbers spanning 5 decades to date was evaluated and analyzed.

## 3. Results

### 3.1. Epidemiology

Uveitis diagnosis among elderly patients older than 60 years of age was considered uncommon. The previous impression about the common age of presentation in uveitis was based on epidemiology studies in the last century. Darrell et al. in 1962 demonstrated that only 14% of uveitis patients were considered elderly (>60 years of age) [1]. However, this trend is changing especially in the developed countries in the last two decades [4, 5].

Reviewing the published uveitis epidemiology studies in the last 50 years, we find that the number of published reports has almost doubled after the year 2000 (15 reports published between 1960 and 1999 [1, 8–20] versus 26 reports published after 2000 [4, 5, 16, 20–43]). Specifically, much more reports have been published from developing countries after the year 2000 (3 reports were published prior to 2000 [10, 15, 44] compared to 12 reports after 2000 [3, 21, 22, 24–26, 30, 32, 34, 35, 39, 41]).

Studying the total data from studies in the past 5 decades revealed that mean age at presentation is 38.0 ± 5.0 (range: 29.0–46.5) (Table 1). Comparing the mean age at presentation between developed versus developing countries shows that mean age at presentation in developed countries is more than in developing countries: 40.6 ± 4.7 (range: 33.8–46.1) versus 34.4 ± 2.7 (range: 29.0–39.9), respectively. If we compare data published before year 2000 to that published after, we find that there is no difference in the mean age at presentation (38.3 versus 37.8) (Table 1).

Also, comparing the mean proportion of elderly patients among total uveitis patients between developed versus developing countries revealed that this group of patients occupies a large number of patients in most of the epidemiology studies in developed countries with mean percentile of 18.6% ± 5.7 (range: 13.6–29.9%). However, the mean percentile of elderly patients among uveitis patients in developing countries is limited to 7.2% ± 2.2 (range: 3.0–10.2%) only. The percentile of elderly patients increased in reports from developing countries from 5.7 prior to year 2000 to 8.4% after. This might be due to increased reporting (Table 2).

This discrepancy in the mean percentile of elderly patients among total uveitis patients between developed and developing countries may have two potential explanations. First, most of the developed countries are considered according to the latest WHO report as aging countries with an increasing proportion of elderly population. Hence, more elderly patients with ocular inflammation are expected to be examined. Second, with an increasing awareness of the recommended screening tests and an improvement in the health care systems in the developed countries, more patients are expected to be examined and to be followed up in these countries including elderly patients.

Most of the major uveitis epidemiology studies demonstrated no gender preference or slight preference toward females. However, reviewing the data of eight epidemiology studies reporting the gender preference among elderly patients revealed that uveitis among elderly females is significantly more than elderly males (F : M = 2.0) (*P* = 0.021) [11, 12, 14, 27, 28, 38, 42, 45]. The ratio decreased from 2.5 before 2000 to 1.7 after.

### 3.2. Most Common Location and Diagnosis

Generally, the most common location of uveitis worldwide is anterior uveitis. Similarly, most of the uveitis epidemiology studies in the elderly reported anterior uveitis as the most common location of this disease at presentation (507 out of 823 cases), followed by panuveitis (129 out of 823 cases) and posterior uveitis (112 out of 823 cases) (Table 3).

Also, the most common diagnosis of uveitis among elderly was reported as idiopathic uveitis in five out of ten reports. However, acute anterior uveitis was found to be the most common diagnosis in three studies. In addition, herpetic induced uveitis was reported as the second or the third most common cause of uveitis in elderly in seven out of ten studies. Most of the reviewed epidemiology studies based their diagnosis of HSV, VZV, and CMV anterior uveitis on careful ocular and medical history in combination with positive antibody titers or DNA detection in the intraocular fluid using PCR method [17, 21, 25, 34, 42, 43]. However, two studies based their diagnosis of herpetic and CMV uveitis on the clinical findings only [26, 39]. Ocular tuberculosis, toxoplasmosis, birdshot, and lymphoma were all reported in the elderly population after the year 2000. That might be due to the increased reporting from developing countries after the turn of the century or to increased awareness and advances in diagnostic modalities of some conditions such as ocular tuberculosis and lymphoma (Table 4).

Interestingly, none of the available epidemiology studies found that masquerades or neoplasm was a common cause of uveitis among elderly patients. In addition, only 4 of the reviewed papers reported neoplasms as a cause of uveitis in this age group (12 out of 261 cases) [10, 15, 18, 20]. Primary or metastatic ocular lymphoma can present with wide spectrum of age distribution, including young patients [46–49]. Actually, our current epidemiology studies demonstrated that ocular lymphoma was one of the common etiologies of ocular inflammation in only one study [16]. As the disease progresses, it can mimic the inflammation of uveitis and is often inappropriately treated with corticosteroids. In primacy intraocular lymphoma, definitive diagnosis requires identification of malignant lymphoid cells from ocular tissue or CSF. Several techniques exist to obtain the required tissue, including aqueous aspiration, diagnostic vitrectomy, and diagnostic retinal or choroidal biopsy [46–49].

Only one study reported the role of diagnostic pars plana vitrectomy (PPV) in our currently reviewed epidemiology reports. Chatzistefanou et al. showed that, of 19 cases that underwent diagnostic PPV, 2 cases had ocular lymphoma and 3 cases were diagnosed with intraocular infection [17]. In addition, many reports previously demonstrated the importance of diagnostic PPV in cases of uveitis with unknown etiology. It was shown that PPV is a helpful tool with diagnostic yield ranging from 14.3% to 61.5% of uveitis with unknown causes [50–58].

### 3.3. Associated Comorbidities

Similarly to the younger patients, autoimmune diseases like sarcoidosis, inflammatory bowel diseases, and insulin dependent diabetes mellitus were reported as common comorbidities among the elderly patients with uveitis [20]. Noninsulin dependent diabetes mellitus was listed by some reports as a common comorbidity among elderly patients with uveitis [17, 20]. However, further data analysis by Chatzistefanou et al. failed to reproduce any specific correlation between diabetes type II and uveitis among the elderly in their study [17].

## 4. Discussion

### 4.1. Immune System Changes among the Elderly

Uveitis is an inflammatory process affecting one or more of the eye globe layers. Understanding the mechanism of work of the immune system and the changes associated with age is key to explaining the differences observed in uveitis demographics among the elderly patients. It is known that the elements of the innate and the acquired immune system undergo changes with age. This process is labeled immunosenescence [59]. Studies suggest that lymphocytes' ability for proliferation and activation is decreased with age. T-cells among other elements of the immune system are believed to play a major role in ocular inflammatory processes. In particular, Th1 mediated response by T-cells gets altered with age secondary to irregular cell-cell interactions that take place at many levels including the antigen presenting cells (APC) [2, 59]. This explains the increased rate of infection by certain pathogens in the elderly, like influenza, Herpes, and tuberculosis [60, 61]. As for the paradoxical increase in the number of antibodies produced by B-cells, they are found to be less functional and less specific [59]. The weaker immune system among the elderly renders them more prone to develop uveitis secondary to infectious causes. This was obviously translated in our paper by an increase in the proportion of patients diagnosed with herpetic uveitis. It should be noted also that a weak immune reaction in the elderly alters the classical clinical presentation of a disease. Patients with Varicella Zoster ophthalmicus might not manifest the typical skin vesicles. Similarly, endophthalmitis in elderly might not develop a severe inflammatory reaction except late in the course of the disease.

### 4.2. Interpretation of Our Results

In our study, we have found that the proportion of elderly (aging 60 years or more) among the uveitis patients is more in the developed countries (18.6%) compared to the developing countries (7.2%). This observation can be mainly explained by the increasingly aging populations of the developed countries compared to the younger societies in the developing countries [62]. Even with the increased reporting from the developing countries after the year of 2000, the proportion of the elderly among the uveitis patients in these areas did not exceed 8.5%. This suggests that the fewer number of reports published in the developing countries is not responsible for the difference mentioned above between the developing countries and the developed countries.

Extrapolating from the bigger proportion of elderly among the uveitis patients in the developed countries, it is expected that the mean age at presentation of patients with uveitis should be more in those countries compared to the developing countries. In our paper, we found a mean age of presentation of 40.6 in the developed countries, compared to 34.4 in the developing countries.

In our paper, we report that de novo uveitis attacks among the elderly have a female predominance. However female predominance was not always reproducible in all the reports included in our review.

It was also mentioned in our statistics that neoplasms are uncommonly reported. It should be noted however that masquerades occur more frequently among the elderly population and in case they are suspected, an extensive workup is sometimes required in order to rule them out including PPV and tissue analysis [63].

### 4.3. Diagnosing Uveitis among the Elderly

Uveitis affects all age groups, but the differential diagnosis steers toward specific entities with each age category. Seronegative spondyloarthropathies very rarely manifest as uveitis de novo in elderly patients. On the other hand, masquerades are more common among the elderly and high level of suspicion should be kept in mind while ruling them out.

Location of uveitis is the main subcategory used to differentiate the major entities of uveitis. Clinicopathologic picture, onset and course of the disease, signs and symptoms, and review of system are other useful categories that are employed to narrow down the differential diagnosis.

**Figure 1:**
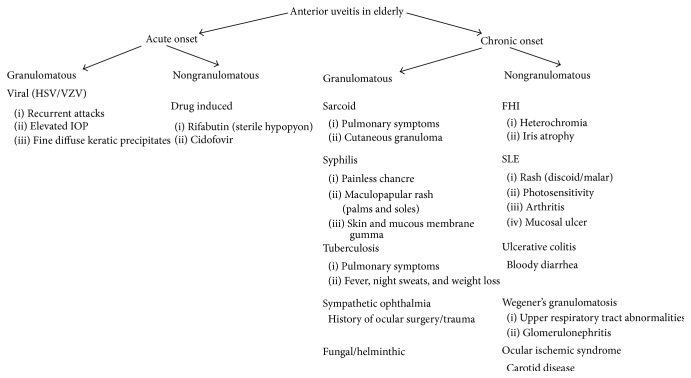
Differential diagnosis of anterior uveitis in elderly patients. HSV: Herpes Simplex virus. VZV: Varicella Zoster virus. VKH: Vogt Kayanagi Harada syndrome. FHI: Fuch's heterochromic iridocyclitis. SLE: Systemic Lupus Erythematosus.

**Figure 2:**
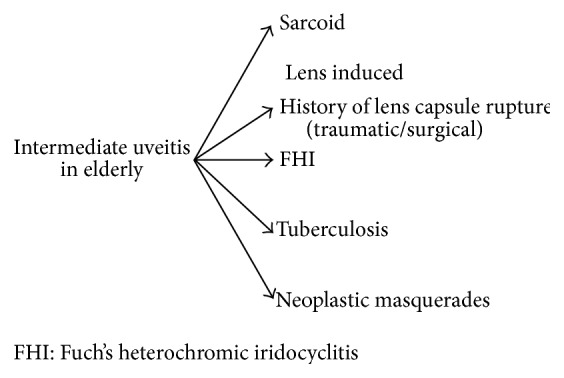
Differential diagnosis of intermediate uveitis in elderly patients. FHI: Fuch's heterochromic iridocyclitis.

**Figure 3:**
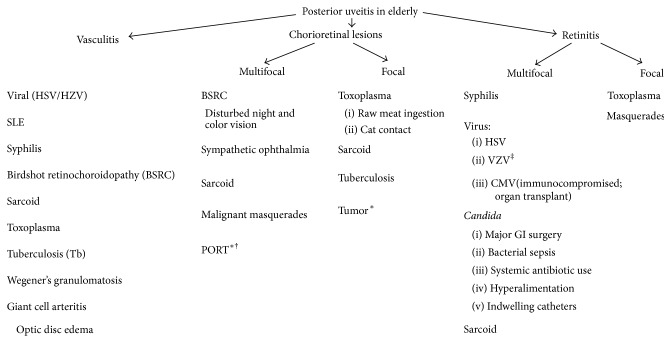
Differential diagnosis of posterior uveitis in elderly patients. usually occur ∗* without* vitritis. ^†^Elderly and immunocompromised elderly may manifest atypical lesions (large, multiple, and bilateral). ^‡^Immunocompromised elderly may get progressive outer retinal necrosis (PORN). PORT: punctate outer retinal toxoplasmosis. CMV: Cytomegalovirus.

Figure 1 depicts the entities that would present as anterior uveitis in the elderly. Segregation was based on the onset of the disease, presence or absence of granulomatous reactions, and some distinguishing feature. Special attention should be given to ischemic syndrome secondary to carotid disease in the elderly. Furthermore, viral anterior uveitis is a significant entity in this population. The altered immunologic status of the elderly, generally characterized by a relative cellular immune deficiency, may mask some of the clinical findings warranting definitive diagnostic workup in suspected cases of HSV, VZV, and CMV uveitis using viral PCR of the aqueous humor tap.  Figure 2 lists the limited entities of intermediate uveitis in the elderly. It should be noted that pars planitis and multiple sclerosis are not part of the differential listed because these entities are rarely reported in patients older than 60 years of age. It is also worth noting that lens/IOL-induced uveitis is much more common in the elderly cataractous lens. Fuch's heterochromic iridocyclitis and lens-induced uveitis may present as intermediate or as panuveitis.  Figure 3 delineates the entities that present as de novo posterior uveitis in the elderly. Many of them are characterized by an extensive inflammation and may present as panuveitis like sympathetic ophthalmia and birdshot chorioretinopathy. Of particular interest, endophthalmitis is more common among the elderly. It presents as panuveitis. One must keep in mind that immunocompromised elderly have a weaker immune system and hence a milder form of inflammation should be anticipated. Sarcoidosis, tuberculosis, and syphilis can mimic any form of uveitis, including panuveitis (Figures 1–3). Furthermore, in elderly patients with altered immunologic status which may mask some of the clinical findings and with the availability of the less invasive 25-gauge vitrectomy, diagnostic PPV may be very helpful in determining the unknown etiology of uveitis in the elderly particularly ruling out infections or cancers.

### 4.4. Treating Uveitis among the Elderly

As in uveitis of any age, treatment is directed to the specific etiology. In case of an infectious etiology, specific anti-infectious medications are given according to the specific organism. While in cases of inflammatory/autoimmune uveitis, treatment with corticosteroids and immune suppressive therapy is instituted. On the other hand, malignant conditions such as lymphoma are treated with local/systemic chemotherapy. However, care should be taken in this elderly population to monitor for drug side effects especially hepatic and renal toxicity and bone marrow suppression, as this age group tends to have other comorbidities and chronic diseases such as osteoporosis, diabetes, and hypertension that puts them at added risks of drug induced complications. Furthermore, drug interactions should be taken into account given the higher likelihood in this age group that the patient will be on medications for other diseases.

## 5. Conclusion

Uveitis in the elderly represents 14.7% of the total uveitis population reported worldwide. It is more commonly reported in the developing world representing 18.6% of the uveitis population. The percentage of elderly uveitis patients is increasing with recent reports after the year 2000. Moreover, there is a female preponderance in the elderly uveitis group; however, this ratio is decreasing with recent reports. Anterior uveitis is the most common presentation worldwide and infections such as tuberculosis and toxoplasmosis are increasingly being reported in recent years. Furthermore, neoplasms do not constitute a sizeable proportion of uveitis in the elderly but need to be ruled out as a masquerade syndrome in this age group.

The socioeconomic burden of this disease relies on the fact that it affects mainly the population of productive ages (aging 20 to 60) [64]. Little is mentioned about uveitis as being a cause of blindness among the elderly. But more recently, it was reported that it is underestimated and that such a rare disease among the elderly has a lot of socioeconomic impact especially with the continuously aging societies in the developed countries [65].

## Figures and Tables

**Table 1:** Mean age at presentation in uveitis epidemiology study.

Study name	Total number of patients	Mean age
Lebanon 2014 [21]	209	36
Iran 2014 [22]	2016	33.8
Italy 2010 [23]	1065	41
Saudi Arabia 2010 [24]	351	39.9
Colombia 2009 [25]	693	31.7
Saudi Arabia 2009 [26]	488	38
Japan 2009 [27]	834	46.1
Japan 2009 (2) [28]	1240	44.1
Germany 2009 [29]	1916	35
Turkey 2008 [30]	761	35.4
Thailand 2008 [31]	200	38
Tunisia 2007 [32]	219	34
China 2005 [33]	1752	33.8
Turkey 2005 [34]	300	35.7
Iran 2004 [35]	544	33.1
Japan 2003 [36]	189	45
USA 2003 [37]	853	46.1
China 2003 [38]	160	41.1
Saudi Arabia 2002 [39]	200	34
Italy 2001 [40]	655	44.3
Cameron 2001 [41]	38	33.9
India 2000 [3]	308	32.5
Italy 1996 [8]	1417	30.7
UK 1996 [9]	712	39.9
Sierra Leone 1996 [10]	93	36
Japan 1997 [11]	551	46.5
Switzerland 1994 [12]	558	44
Holland 1992 [13]	881	42
Portugal 1990 [14]	450	36
Japan 1997 [11]	407	40.7
Nigeria 1977 [15]	1987	29

Mean		38.0

**Table 2:** Proportion of elderly patients in uveitis epidemiology studies among developed countries.

Developed countries		
Name	Total number	Elderly patients percentile
China 2012 [5]	5866	1538 (26.2%)
Italy 2010 [23]	1065	206 (20.3%)
Japan 2009 [27]	843	224 (26.8%)
Japan 2009 (2) [28]	1240	190 (15.3%)
Germany 2009 [29]	1916	(16%)
China 2003 [38]	160	28 (17.5%)
Italy 2001 [40]	655	89 (13.6%)
France 2000 [16]	125	19 (15.2%)
Switzerland 1998 [17]	558	151 (27%)
Australia 1994 [18]	245	37 (15%)
USA 1998 [17]	1328	138 (10.4%)
Holland 1992 [13]	865	182 (20%)
Japan 1997 [11]	551	165 (29.9%)
Italy 1996 [8]	1417	228 (16.1%)
Finland 1994 [18]	1122	191 (17%)
Finland 1975 [19]	653	89 (13.6%)
USA 1962 [1]		14

Developing countries		
Study	Total number	Elderly patients percentile

Lebanon 2014 [21]	209	18 (9%)
Saudi Arabia 2009 [26]	488	50 (10.2%)
Colombia 2009 [25]	693	55 (7.9%)
Turkey 2008 [30]	761	50 (6.6%)
Tunisia 2007 [32]	219	36 (7.6%)
Saudi Arabia 2002 [39]	200	18 (9%)
India 2009 [44]	1273	82 (6.4%)
Nigeria 1977 [15]	1987	60 (3.0%)
Lebanon 2014 [21]	209	18 (9%)
Saudi Arabia 2009 [26]	488	50 (10.2%)
Colombia 2009 [25]	693	55 (7.9%)
Turkey 2008 [30]	761	50 (6.6%)

**Table 3:** Most common location of uveitis in elderly patients.

Study name (*n*)	Anterior (%)	Posterior (%)	Panuveitis (%)	Intermediate (%)
Lebanon 2014 (*n* = 18) [21]	9 (50)	1 (5.5)	8 (4.5)	0
UK 1994 (71) [20]	44 (62)	7 (9.9)	14 (19.7)	6 (8.5)
Saudi Arabia 2009 (*n* = 50) [26]	39 (78)	2 (4)	5 (10)	4 (8)
Finland 1994 (*n* = 191) [18]	187 (97.9)	1 (0.1)	2 (1.04)	1 (0.1)
France 2000 (*n* = 19) [16]	5 (26.3)	6 (31.6)	8 (42.1)	0
Japan 2005 (*n* = 82) [42]	30 (36.6)	24 (29.2)	16 (19.5)	2 (2.4)
France 2003 (*n* = 80) [43]	34 (42.5)	18 (22.5)	20 (25)	8 (10)
Saudi Arabia 2002 (*n* = 20) [39]	13 (72.2)	2 (11.1)	2 (11.1)	1 (5.6)
Italy 2010 (*n* = 206) [23]	100 (48.6)	48 (23.3)	54 (26.2)	4 (19.4)
Finland 1975 (*n* = 86) [19]	80 (93)	3 (3.4)	3 (3.4)	0

Total	541	112	129	26

**Table 4:** Most common diagnosis of uveitis in elderly patients.

Study	Most common	Second common	Third common
Lebanon 2014 [21]	Idiopathic uveitis (5/18)	HSV (5/18)	TB (3/18)
Colombia 2009 [25]	Toxoplasmosis (10/55)	Idiopathic uveitis (9/55)	HSV (5/55)
Saudi Arabia 2009 [26]	AAU (14/51)	HSV(8/51)	TB (7/51)
Turkey 2005 [34]	Idiopathic uveitis (34/50)	HSV (3/50)	Sarcoidosis (2/50)
Japan 2005 [42]	Idiopathic uveitis (53/82)	Sarcoidosis (9/82)	HSV (7/82)
France 2003 [43]	Idiopathic uveitis (40/80)	HSV/VZV (10/80)	Birdshot (10/80)
Saudi Arabia 2002 [39]	AAU (5/18)	TB (5/18)	HSV (3/18)
France 2000 [16]	Idiopathic uveitis (5/19)	Sarcoidosis (3/19)	Lymphoma (2/19)
USA 1998 [17]	Idiopathic uveitis (43/138)	HSV (16/138)	Sarcoidosis (11/138)
Finland 1994 [18] (*n* = 191)	AAU (118/191)	Idiopathic uveitis (21/191)	Sarcoidosis (2/191)
UK 1994 [20]	Idiopathic uveitis (55/71)	IDDM 5/71	Sarcoidosis (3/71)

AAU: acute anterior uveitis, HSV: herpetic simplex virus, TB: tuberculosis, VZV: Varicella Zoster virus, IDDM: insulin dependent diabetes mellitus.
